# Supplementary material for: Small herbivores and abiotic heterogeneity promote trait variation of a saltmarsh plant in local communities
Source: PeerJ. 2021 Dec 22;9:e12633. doi: 10.7717/peerj.12633 (PMC8710046; doi:10.7717/peerj.12633)
Supplement: Supplemental Information 1 [file peerj-09-12633-s001.pdf]

Online supporting text

Grazing pressure

To count droppings from hares and geese, we set up a line transect adjacent to the exclosures at each successional stage in 2000 and 2016. Each line transect consisted of 20 plots (4 m^2), with at least 10 m distance between each other (Note that exact position of line transects and plots therein might differ between 2000 and 2016, details in Kuijper & Bakker (2005)). We counted and removed droppings from hares and geese within plots every two or three weeks for the whole year both in 2000 (October 1999 to September 2000) and 2016 (May 2016 to April 2017). Biomass consumed is calculated as FM / (1- DE). FM: total dropping mass (0.85 g per goose dropping; 0.15 g per hare dropping); DE: digestive efficiency (35 % dry matter digestibility for geese; 65 % for hares). Values for dropping mass and digestive efficiency were obtained from Van der Wal et al. (1998). See Table S1 for results. Analysis of variance showed that only the successional stage had significant effects ($F_{1,76}$ = 6.63, P = 0.0119), suggesting that grazing pressure at the early successional stage was higher than the intermediate successional stage in both 2000 and 2016.

Specific leaf area and leaf dry matter content

Although specific leaf area and leaf dry matter content were not measured in the standard way (Pérez-Harguindeguy et al., 2016), a similar method was used in a previous study in this system (Veeneklaas et al., 2011). Note that leaf dry matter content was only measured in 3, 5, 4, and 4 plots in the grazed and ungrazed treatment at the early and intermediate successional stage, respectively. This is because we needed to bring all samples and measure the fresh weight of each leaf sample and the whole plants in the field station. We also need to do this quickly, otherwise, water will be lost in the plant tissues, which may bias estimation of fresh weight. Due to lack of manpower, we measured fresh leaf weight for around half of the randomly selected plots.

An example of SEM

The sem and the specification of direct and indirect effects of small herbivores on ITV in height at the intermediate stage

```
Mod.h = '
##direct links to height
height~c1*cv.cl
height~e1*cv.el
height~gr1*genotypic.richness
height~gd1*genotypic.diversity
height~g1*grazing ## direct effects of herbivores
## grazing effects on abiotic and genotypes
cv.cl ~ c2*grazing
cv.el ~ e2*grazing
genotypic.richness ~ gr2*grazing
genotypic.diversity~ gd2*grazing
##indirect effect
cv:=c1*c2 ## through variation in clay thickness
ev:=e1*e2 ## through variation in elevation
```

```
cvev:=c1*c2+e1*e2 ## through the effects of variations in clay thickness and elevation
gr:=gr1*gr2 ## through genotypic richness
gd:=gd1*gd2 ## through genotypic diversity
grgd:= gr1*gr2+ gd1*gd2 ## through both genotypic richness and diversity
## total effect
total:=g1+(c1*c2)+(e1*e2)+( gd1*gd2)+(gr1*gr2)
```

model output $ Defined Parameters:

	Estimate	Std.Err	z-value	P(>|z|)	Std.lv	Std.all
cv	-0.006	0.006	-0.934	0.350	-0.006	-0.102
ev	-0.004	0.005	-0.724	0.469	-0.004	-0.067
cvev	-0.010	0.008	-1.181	0.238	-0.010	-0.169
gr	0.001	0.003	0.430	0.667	0.001	0.026
gd	0.001	0.007	0.156	0.876	0.001	0.019
grgd	0.003	0.008	0.328	0.743	0.003	0.045
total	0.026	0.014	1.905	0.057	0.026	0.454

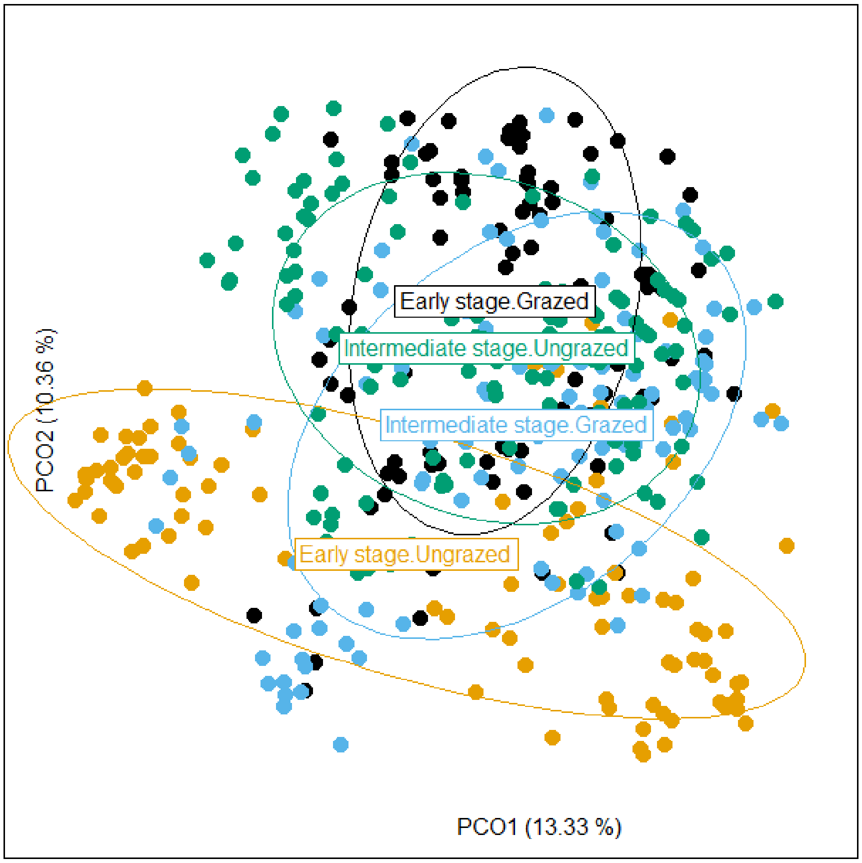

Fig. S1. PCoA-plot of genetic distances of individuals of *Elytrigia atherica*. The population from the ungrazed treatment substantially differentiated from that of the grazed at early successional stage. The centroids of the grazed and ungrazed at the early and intermediate

successional stage are shown. The ellipses denote 95 % bivariate confidence intervals. The percentages denote the proportion of variance explained by PCoA axes.

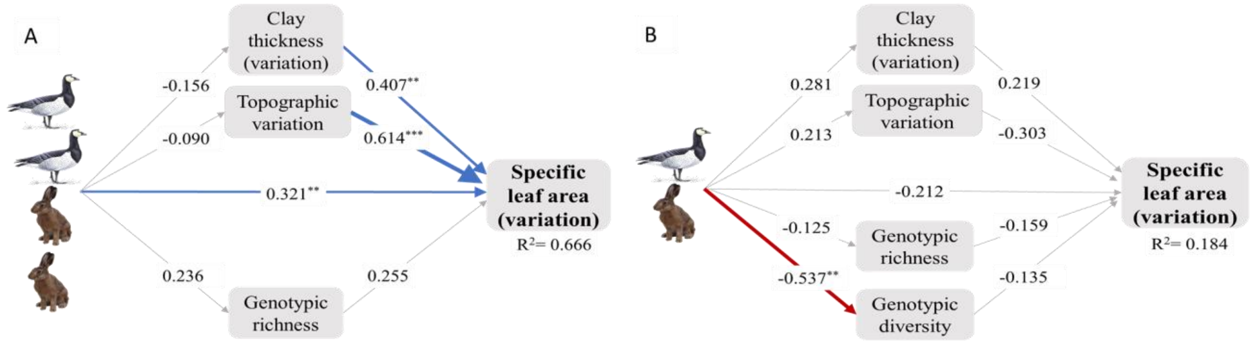

Fig. S2. Intraspecific trait variation (ITV) of the dominant plant *Elytrigia atherica* and the direct and indirect effects of small herbivores on ITV in local communities at the early (A) and intermediate successional stage (B). The direct effects, indirect effects through genotypes, indirect effects through abiotic variations, and total effects at the early and intermediate stages are 0.321^{**}, 0.06, -0.119, 0.262, and -0.212, 0.092, -0.003, -0.122, respectively. Model fit the data well (at the early successional stage: $\chi^2 = 4.409$, df = 3, N = 14, $p > 0.05$; at the intermediate successional stage: $\chi^2 = 6.559$, df = 6, N = 14, $p > 0.05$). Variance explained for clay thickness (variation), topographic variation, and genotypic richness for models at the early successional stage are 0.024, 0.008, and 0.056, respectively. Variance explained for clay thickness (variation), topographic variation, genotypic richness, and genotype diversity for models at the intermediate successional stage are 0.079, 0.045, 0.016, and 0.289, respectively. Number of hares and geese indicate the abundance of small herbivores such that the early successional stage had higher grazing pressure (indicated by two hares and two geese) relative to the intermediate stage (indicated by one hare and one goose). Boxes are measured variables. Arrows denote unidirectional relationships among variables. Blue arrows are significant positive relationships, red arrows are significant negative relationships, and grey arrows show non-significant relationships. The width of the arrows indicates the strength of the pathways. The values on the arrows denote standardized path coefficients. Asterisks indicate significant paths: * $p < 0.1$; ** $p < 0.05$; *** $p < 0.001$.

Table S1. Biomass consumed by hares and geese at the early and intermediate successional stage in 2000 and 2016. Droppings were the means (± 1 se) of the 20 4-m^2 plots, each with the summed whole year droppings, at each successional stage.

Succession stage	Year	N	Droppings (hares/geese)	Biomass consumed
Early stage	2000	20	84.25/64.95	30.26 ± 4.371
	2016	20	134.05/37.65	26.67 ± 5.277
Intermediate stage	2000	20	39.75/31.60	14.59 ± 1.939
	2016	20	52.00/50.40	22.05 ± 3.374

Table S2 community properties in the permanent plots (2 m × 2 m) of the grazed and ungrazed area at the early and intermediate successional stage in 2016. Shown are the means ± 1 se of 8 permanent plots for total cover, number of species, species with cover >=10 % (*Elytrigia atherica* is shown even its cover <10%) in the grazed and ungrazed treatments.

Successional stage	Grazing	Community properties	Cover (%)/number of species
Early	Grazed	Total coverage	104.81 ± 1.55
		Species richness	6.25 ± 0.31
		Artemisia maritima	56.25 ± 4.2
		Festuca rubra	40 ± 1.89
		Elytrigia atherica	2.81 ± 1.85
	Ungrazed	Total coverage	102.69 ± 1.23
		Species richness	2.75 ± 0.25
		Elytrigia atherica	99.75 ± 0.25
Intermediate	Grazed	Total coverage	106.31 ± 4.77
		Species richness	7.5 ± 1.16
		Artemisia maritima	27 ± 10.85
		Elytrigia atherica	14.25 ± 5.8
		Festuca rubra	11.88 ± 5.42
		Aster tripolium	11.25 ± 4.41
	Ungrazed	Total coverage	99.5 ± 2.8
		Species richness	6.5 ± 1.02
		Elytrigia atherica	61.56 ± 13
		Atriplex portulacoides	21.5 ± 8.97

Table S3 Model summary for the treatment effects on trait means and variations at the early and intermediate successional stages.

Variables	Successional stage	Terms	Df	Sum Sq	Mean Sq	F value	Pr(>F)
height_avg	Early	grazing	1	1372.43	1372.43	18.76	0
	Early	Residuals	12	877.68	73.14	#N/A	#N/A
	Intermediate	grazing	1	400.85	400.85	27.18	0
	Intermediate	Residuals	12	176.99	14.75	#N/A	#N/A
aboveground.biomass_avg	Early	grazing	1	0.51	0.51	55.95	0
	Early	Residuals	12	0.11	0.01	#N/A	#N/A
	Intermediate	grazing	1	0.27	0.27	29.4	0
	Intermediate	Residuals	12	0.11	0.01	#N/A	#N/A
flower_avg	Early	grazing	1	0.28	0.28	18.83	0
	Early	Residuals	12	0.18	0.01	#N/A	#N/A
	Intermediate	grazing	1	0.18	0.18	4.76	0.05
	Intermediate	Residuals	12	0.46	0.04	#N/A	#N/A
specific.leaf.area_avg	Early	grazing	1	33.37	33.37	0.26	0.62
	Early	Residuals	12	1545.49	128.79	#N/A	#N/A
	Intermediate	grazing	1	833.35	833.35	5.49	0.04
	Intermediate	Residuals	12	1820.59	151.72	#N/A	#N/A
leaf.dry.matter.content_avg	Early	grazing	1	0.01	0.01	0.4	0.55
	Early	Residuals	6	0.22	0.04	#N/A	#N/A
	Intermediate	grazing	1	0	0	0.26	0.63
	Intermediate	Residuals	6	0.02	0	#N/A	#N/A
height_cv	Early	grazing	1	0.02	0.02	3.89	0.07
	Early	Residuals	12	0.07	0.01	#N/A	#N/A
	Intermediate	grazing	1	0	0	3.05	0.11

	Intermediate	Residuals	12	0.01	0	#N/A	#N/A
aboveground.biomass_cv	Early	grazing	1	0.01	0.01	0.62	0.45
	Early	Residuals	12	0.2	0.02	#N/A	#N/A
	Intermediate	grazing	1	0.01	0.01	1.24	0.29
	Intermediate	Residuals	12	0.05	0	#N/A	#N/A
flower_cv	Early	grazing	1	8.21	8.21	13.42	0
	Early	Residuals	12	7.34	0.61	#N/A	#N/A
	Intermediate	grazing	1	1.84	1.84	1.56	0.23
	Intermediate	Residuals	12	14.15	1.18	#N/A	#N/A
specific.leaf.area_cv	Early	grazing	1	0	0	0.98	0.34
	Early	Residuals	12	0.04	0	#N/A	#N/A
	Intermediate	grazing	1	0	0	0.19	0.67
	Intermediate	Residuals	12	0.09	0.01	#N/A	#N/A
leaf.dry.matter.content_cv	Early	grazing	1	0.07	0.07	0.16	0.71
	Early	Residuals	6	2.83	0.47	#N/A	#N/A
	Intermediate	grazing	1	0.15	0.15	1.06	0.34
	Intermediate	Residuals	6	0.83	0.14	#N/A	#N/A
